# Placental Structure in Preterm Birth Among HIV-Positive Versus HIV-Negative Women in Kenya

**DOI:** 10.1097/QAI.0000000000001871

**Published:** 2018-09-27

**Authors:** Moses M. Obimbo, Yan Zhou, Michael T. McMaster, Craig R. Cohen, Zahida Qureshi, John Ong’ech, Julius A. Ogeng’o, Susan J. Fisher

**Affiliations:** Departments of * Human Anatomy; and; † Obstetrics and Gynecology, University of Nairobi, Nairobi, Kenya;; ‡ Department of Obstetrics, Gynecology and Reproductive Sciences, Center for Reproductive Sciences, University of California San Francisco, San Francisco, CA;; § The Eli & Edythe Broad Center for Regeneration Medicine and Stem Cell Research, University of California San Francisco, San Francisco, CA;; ║ Department of Obstetrics, Gynecology and Reproductive Health, Bixby Center for Reproductive Health, University of California San Francisco, San Francisco, CA;; ¶ Department of Cell and Tissue Biology, University of California San Francisco, San Francisco, CA;; # Department of Obstetrics and Gynecology, Kenyatta National Hospital, Nairobi, Kenya; and; ** Department of Anatomy, University of California San Francisco, San Francisco, CA

**Keywords:** preterm birth, term birth, placenta, HIV, ART

## Abstract

**Background:**

Preterm birth (PTB) is a major cause of infant morbidity and mortality in developing countries. Recent data suggest that in addition to Human Immunodeficiency Virus (HIV) infection, use of antiretroviral therapy (ART) increases the risk of PTB. As the mechanisms remain unexplored, we conducted this study to determine whether HIV and ART were associated with placental changes that could contribute to PTB.

**Setting:**

We collected and evaluated placentas from 38 HIV-positive women on ART and 43 HIV-negative women who had preterm deliveries in Nairobi, Kenya.

**Methods:**

Anatomical features of the placentas were examined at gross and microscopic levels. Cases were matched for gestational age and compared by the investigators who were blinded to maternal HIV serostatus.

**Results:**

Among preterm placentas, HIV infection was significantly associated with thrombosis (*P* = 0.001), infarction (*P* = 0.032), anomalies in cord insertion (*P* = 0.02), gross evidence of membrane infection (*P* = 0.043), and reduced placental thickness (*P* = 0.010). Overall, preterm placentas in both groups were associated with immature villi, syncytial knotting, villitis, and deciduitis. Features of HIV-positive versus HIV-negative placentas included significant fibrinoid deposition with villus degeneration, syncytiotrophoblast delamination, red blood cell adhesion, hypervascularity, and reduction in both surface area and perimeter of the terminal villi.

**Conclusions:**

These results imply that HIV infection and/or ART are associated with morphological changes in preterm placentas that contribute to delivery before 37 weeks. Hypervascularity suggests that the observed pathologies may be attributable, in part, to hypoxia. Further research to explore potential mechanisms will help elucidate the pathways that are involved perhaps pointing to interventions for decreasing the risk of prematurity among HIV-positive women.

## INTRODUCTION

Preterm birth (PTB) affects approximately 10% of all pregnancies worldwide and leads to early infant morbidity, mortality, and chronic childhood diseases.^1^ Multiple factors increase a woman's risk of PTB, including advanced maternal age, short intergestational interval, chronic illnesses such as hypertension and diabetes, low maternal socioeconomic status, and bacterial vaginosis.^2,3^ However, the biological mechanisms underlying PTB remain poorly understood.^4–6^

In pregnancy complications, placental structure is altered.^7–11^ Although Human Immunodeficiency Virus (HIV) has been associated with adverse obstetric outcomes,^12,13^ the effects of this infection on the structure of preterm placentas remain poorly understood. Some studies have indicated alterations at gross and microscopic levels, including chorioamnionitis, fibrin deposition, formation of syncytial knots, syncytiotrophoblast sloughing, villitis, villous stromal fibrosis, infarction, abnormal villous maturation, deciduitis, and decidual necrosis.^14–18^ By contrast, other investigators have failed to find placental pathological alterations associated with HIV infection in both term and preterm deliveries.^17,19–21^

Although early studies on the association of HIV and antiretroviral therapy (ART) with prematurity have reported conflicting results,^22^ more recent investigations support a relationship.^23,24^ Nevertheless, the data on potential mechanisms by which HIV and ART may increase the risk of PTB are limited. Thus, we conducted this study to determine whether HIV and ART were associated with salient placental pathologies that could contribute to an increased risk of PTB. Identification of these changes is of critical importance to designing studies to explore possible biological mechanisms, and eventually, therapeutic interventions in the setting of HIV and ART in pregnancy aimed at reducing the risk of PTB.

## MATERIAL AND METHODS

### Ethical Considerations

The Kenyatta National Hospital (KNH)/University of Nairobi Ethics and Research Committee reviewed and approved the study protocol. A material transfer agreement was signed between the University of Nairobi and the University of California San Francisco, and authority to transfer material to the United States was obtained from the KNH/University of Nairobi Ethics and Research Committee and the Ministry of Health of Kenya.

### Recruitment and Informed Consent

Trained midwives, one at KNH and the other at Pumwani Maternity Hospital (PMH), identified subjects who met inclusion criteria from the women who were admitted to labor and delivery units between March and August, 2015. Either the principal investigator (M.M.O.) or one of the trained research assistants gave a full explanation of the study and obtained informed consent to enroll.

### Study Population

Placentas were collected from the following groups: (1) 38 HIV-positive women who received ART and had a PTB; (2) 43 HIV-negative women who delivered in the preterm period; (3) 9 HIV-positive women who received ART and delivered at term; and (4) 11 HIV-negative women who delivered at term. Only placentas from women of African descent aged 18–40 years with a singleton live birth and without concomitant medical disorders or obstetric complications such as infections, pre-eclampsia, diabetes, cardiovascular disease, or malnutrition were included in this study. Gestational age was determined by last normal menstrual period and corroborated by an obstetric ultrasound before 16 weeks. PTBs were defined as delivery at <37 weeks of gestation, and term births were defined as delivery ≥37 weeks of gestation.

### Sample Collection

Clinical data and placental samples were collected at 2 sites, KNH and PMH, both located in Nairobi, Kenya. The clinical data included maternal age, gestational age, mode of delivery, and for those that were HIV-positive, recent CD4 counts taken within 6 months of the study period, and their ART status (Table 1). Those on ART were asked to state whether they were adherent to treatment or not. All HIV-positive patients had been on ART preconception. Placentas and the portion of the fetal membranes that were attached to the chorionic plate were obtained aseptically immediately after delivery and assigned a sequential study number (001-101). A macroscopic examination was performed as described below. Six biopsies (designated a–f) of the placentas and fetal membranes were obtained immediately after delivery (2 central from either side of the cord insertion and 4 from peripheral aspects of the placenta at the 12, 3, 6, and 9 o'clock positions) and fixed in 10% neutral buffered formalin according to a standard protocol.^25^ They were then transported to Lancet Laboratories (Nairobi) where the membranes were removed from the placentas and made into rolls; subsequently, the rolls and placental biopsies were dehydrated in increasing concentrations of alcohol (70%–100%), 1 hour per solution. Then, they were cleared in trichloroethane (TCE) for 2 hours and infiltrated with paraffin for 12 hours. Finally, the specimens were embedded in fresh molten paraffin for 12 hours overnight. Blocks, which were sectioned and stained at the University of California San Francisco (UCSF), were subjected to microscopic and morphometric analyses (see below). Stereology was performed at the Department of Human Anatomy, University of Nairobi.

**TABLE 1.**
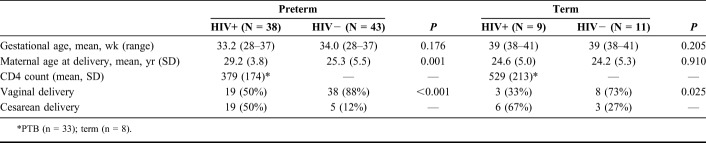
Comparison of Clinical Characteristics and Mode of Delivery of HIV-Positive and HIV-Negative Women Having Preterm and Term Deliveries

### Gross Examination

The weight of the placentas was measured by using a Dual Display-Camry Scale (CE certified) and recorded along with gestational age. The placentas were evaluated at gross and microscopic levels. Grossly, the placentas were examined for areas of infarction or thrombosis, site of cord insertion, shape of the placenta, and color of the membranes and chorionic plate. The length of the umbilical cord from the proximal end (closest to the placental disc) to the distal end, which was transected during delivery, was determined by a flexible tape measure and recorded in centimeters. The length of the umbilical cord segment that remained attached to the newborn was approximately 10 cm. This value was added to the length of the proximal segment. The cord diameter was determined in 3 regions using a digital Vernier caliper and the average was calculated (±SD). In addition, the cords were assessed for both knotting and coiling. The diameter of the placenta was measured, by a flexible tape, and recorded in centimeters. The thickness at the center was determined by piercing with a sharp rod calibrated in millimeters. The shape of the placenta was recorded as one of the following: discoid, annular, circular, horseshoe, or star. Placental membrane color was described as either maroon (normal), green-brown (meconium-stained) or yellow-gray (infected). The information was recorded in a placental pathology form that the Fisher laboratory routinely uses for cases involving obstetrical complications at the University of California San Francisco.^26^

### Microscopic Evaluation

Five micrometer serial sections were cut using a Leitz Wetzlar sledge microtome, floated in warm water, mounted on glass slides, and dried in a hot air oven at 40°C overnight. The sections were stained with Masson's trichrome, picrosirius red, or hematoxylin and eosin (H&E). H&E was used to demonstrate the general histoarchitecture. Masson's trichrome highlighted the connective tissue components. Picrosirius red stained collagen fibers. One block from each placenta was randomly selected for further processing through a simple random sampling technique. Slides were examined under a Leica Automated Systems light microscope connected to a computer and monitor. The team of investigators, who examined the slides, included a placental biologist, 2 anatomists, and a pathologist. The investigators were blinded to the HIV status of the mothers. The general structural organization of the amnion, chorion, and placenta was recorded, and the amount of fibrin deposition was noted. Other features that were scored as described by the Fisher group^26^ included villus degeneration, syncytiotrophoblast delamination, vascularity, red blood cell adhesion to terminal villi, intermediate mature to mature villi, syncytial knotting, villitis, and deciduitis.

### Morphometric Analyses of Terminal Chorionic Villi

The villous structure of placentas from PTBs was further analyzed. Five placentas from the seropositive and 5 placentas from the seronegative preterm groups were randomly selected as were 2 blocks from each case, which were subjected to morphometric analyses at 400×. Tissue sections were examined and digital images were captured, using a Canon color camera interfaced with an Olympus BX41 microscope, from 10 randomly selected areas per slide. Data collection was limited to terminal villi with outlines completely within the microscopic field. Raw images were analyzed by using Image J (Version 1.46; NIH, MD), which enabled calculating the diameter and surface area of terminal villi (μm^2^) as well as estimating the relative number of capillaries in the stromal cores, which was scored per 10 terminal villi as either normal (2–6 capillaries), high (more than 10 capillaries), or low (0 or 1) based on the classification of Altshuler.^27^

### Statistical Analysis

Gross anatomical features, histological findings, and morphometry of preterm placentas were compared among HIV-positive and uninfected cases. Numerical data were analyzed by SPSS, version 20 (Chicago, IL). Mean values, SDs, and frequency tables were compiled using descriptive data. The independent-sample *t* test was used to compare the mean values from the gross examinations by HIV serostatus. Features of the HIV-seropositive versus HIV-seronegative groups were compared by a χ^2^ test. The statistical significance of the morphometric data was determined by 1-way analysis of variance. A *P*-value of <0.05 was considered statistically significant.

## RESULTS

A total of 101 placentas were studied, 81 preterm and 20 term, with median gestational ages of 33 (range 28–37) weeks and 39 (range 38–41) weeks, respectively. Clinical information about these cases is summarized in Table 1. The median maternal age for the preterm delivery group was 27 (range 18–39) years and 24 (range 18–34) years for the mothers with term deliveries. All HIV-positive patients were on ART with 89% having a recent CD4 count assayed within 6 months of the study period. These patients were on the first line Ministry of Health-Kenya recommended treatment of either stavudine or zidovudine plus lamivudine and nevirapine or efavirenz. We did not consider specific treatment regimens as a variable in the analyses. At the time of this investigation, routine viral load monitoring was not performed at the 2 hospitals used for recruitment. Equal numbers of seropositive women with PTBs delivered vaginally and by cesarean section. In this group at term, a cesarean delivery was more common. The seronegative mothers were more likely to have a vaginal birth, either preterm or at term (Table 1).

First, we examined the site of umbilical cord insertion. Of the preterm specimens, 47/81 (58%) were marginal; 28 from seropositive and 19 from seronegative patients. Of the remaining specimens, 33/81 (41%) had eccentric or central cord insertions; 9 were from seropositive and 24 were from seronegative patients (*P* < 0.05). One placenta from a HIV-seropositive patient had a velamentous insertion. In term deliveries, 9/20 (45%) showed marginal cord insertions; 7/9 (78%) were from seropositive cases and 2/9 (22%) were from seronegative cases. Of the remaining 11 term cases with eccentric or central cord insertions, 9/11 (82%) were seronegative and 2/11 (18%) were seropositive (*P* < 0.05).

Fetal membranes from preterm HIV-seropositive patients were evaluated and classified according to color, either “green-brown” due to meconium staining or “yellow-gray,” an indication of bacterial infection. In HIV-seropositive cases, the frequency of abnormal discoloration was 26% (21/81). In HIV-seronegative cases, the frequency was 16% (13/81). The difference between the 2 groups was statistically significant (*P* = 0.022).

Next, we examined placental vasculature. Among the preterm delivery group, thromboses were more common (*P* < 0.001) in placentas from HIV-positive women (31/38; 81.6%) compared with HIV-negative women (18/43; 41.9%). At term, 2/9 (22.2%) placentas from HIV-seropositive women had thromboses, which were not detected in placentas from HIV-seronegative women. When present, the thrombotic lesions were more likely to be subchorionic, sometimes involving the entire placenta. Infarctions were more common in preterm placentas (*P* = 0.032) from HIV-positive (17/38; 44.7%) vs. HIV-negative women (10/43; 23.3%). The same was true in the term birth group (6/9; 66% vs. 2/11; 18%; *P* = 0.028).

With regard to placental morphometry, only placental thickness differed significantly by maternal HIV serostatus among the PTBs (Table 2). Cord diameter and length, as well as placental weight and diameter, were not statistically different between HIV-seropositive and HIV-seronegative cases. None of these measurements were different in the 2 groups at term.

**TABLE 2.**
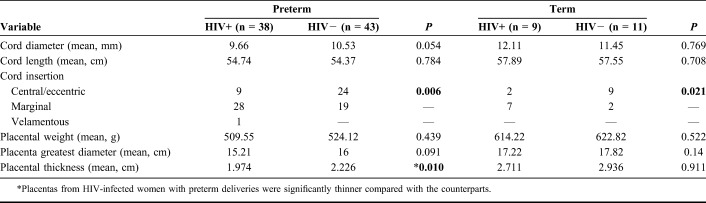
Comparison of the Morphometric Parameters of Placentas From Preterm and Term Deliveries in HIV-Infected and Uninfected Women

We also compared the same umbilical cord and placental variables in the preterm and term groups according to HIV serostatus. As expected, most of the parameters were different in accord with their different gestational ages. In the HIV-seropositive group, cord length and frequency of central/eccentric insertions were not significantly different. In the HIV-seronegative group, there was no significant difference between cord diameter or the frequency central/eccentric insertion. Therefore, the umbilical cord parameters were influenced less by gestational age than the placental variables (Table 3).

**TABLE 3.**
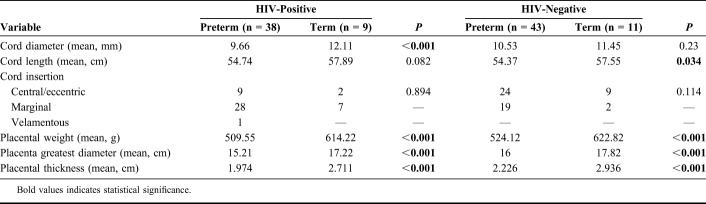
Comparison of Placental Morphometric Parameters (Table 2) by Serostatus

Finally, histologic features of the preterm placentas were scored in a subset of the samples (n = 22/group). The following were significantly different in HIV-positive versus HIV-negative placentas: fibrinoid deposition with villus degeneration [13/22 (59%) vs. 6/22 (27%); *P* = 0.026], syncytiotrophoblast delamination [10/22 (46%) vs. 2/22 (9%); *P* = 0.006], increased red blood cell adhesion to terminal villi [11/22 (50%) vs. 2/22 (9%); *P* = 0.003], and increased number of capillaries [7/22 (32%) vs. 0/22 (0%); *P* < 0.05]. Other histologic features were not different between the preterm groups including intermediate mature to mature villi [18/22 (82%) vs. 16/22 (73%); *P* = 0.355]; syncytial knotting [14/22 (64%) vs. 16/22 (73%); *P* = 0.973]; villitis (11/22 (50%) vs. 9/22 (41%); *P* = 0.403]; and deciduitis [13/22 (59%) vs. 13/22 (59%); *P* = 0.702].

**FIGURE 1.**
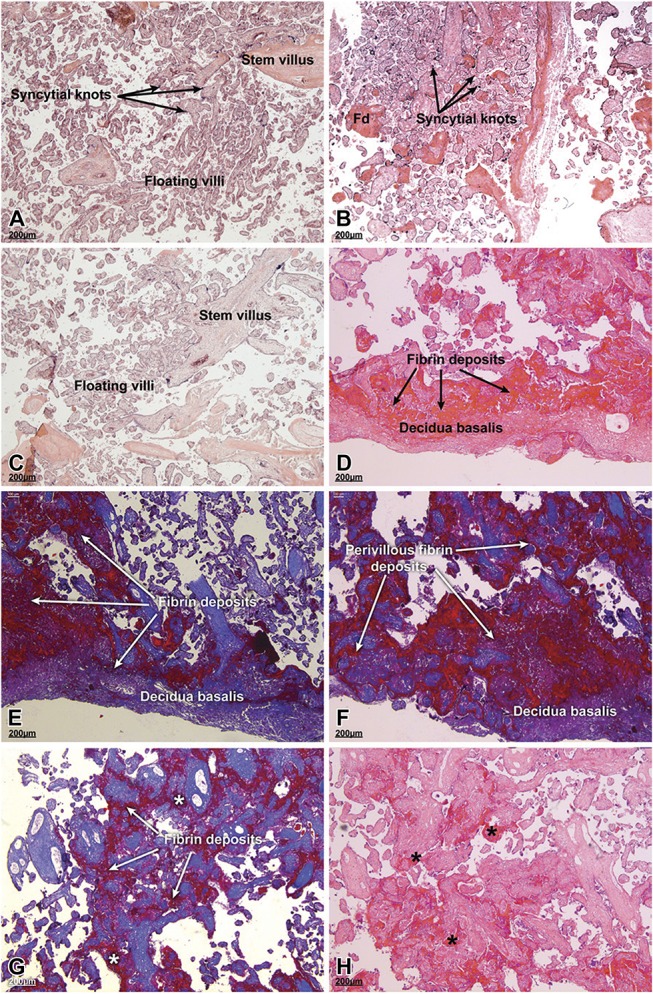
Histology of term and preterm placentas from HIV-positive women on antiretroviral treatment (ART) versus HIV-negative women. The placental biopsies were prepared for paraffin wax embedding, sectioned and stained with either hematoxylin and eosin (A–D and H) or Masson's trichrome (E–G) stains. A, Placenta from a normal HIV-negative pregnancy at term. Note the organization of the floating villi, stem villus, and the syncytial knots. Fibrin material is scant in this section. B, Placenta of a term pregnancy from a HIV-positive woman on ART. Numerous fibrin deposits (Fd) were evident as were syncytial knots, normal in a term placenta. C, Preterm placenta from a HIV-negative woman. The intervillous spaces, which were clear, contained intermediate mature and mature villi. D–H, Preterm placentas from HIV-positive women on ART had obvious fibrin deposition in the perivillous and intervillous regions sometimes obliterating the structure of floating and stem villi. Asterisks in panels G and H show areas of intervillous fibrin deposition. A, 39 weeks; (B) 39 weeks; (C) 33 weeks; (D) 34 weeks; (E) 35 weeks; (F) 34 weeks; (G) 33 weeks; and (H) 30 weeks of gestation. Scale bars, 200 μm.

Figure 1 illustrates the most prominent histologic features of term and preterm placentas from HIV-positive versus HIV-negative women. The sections were stained with either H&E (A–D and H) or Masson's trichrome (E–G). Placentas from a normal HIV-negative pregnancy at term showed the typical microscopic features of floating and stem villi, including syncytial knots (Fig. 1A). Little fibrin deposition was observed. Term placentas from HIV-positive women on ART had similar histologic features except for frequent fibrin deposits (Fig. 1B). Microscopic examination of preterm placentas from HIV-negative women showed numerous intermediate mature to mature villi; fibrin deposits were not observed (Fig. 1C). Preterm placentas from HIV-positive women on ART had extensive fibrin deposits in the perivillous and intervillous regions sometimes obliterating the structure of floating and stem villi (Figs. 1D–H).

**FIGURE 2.**
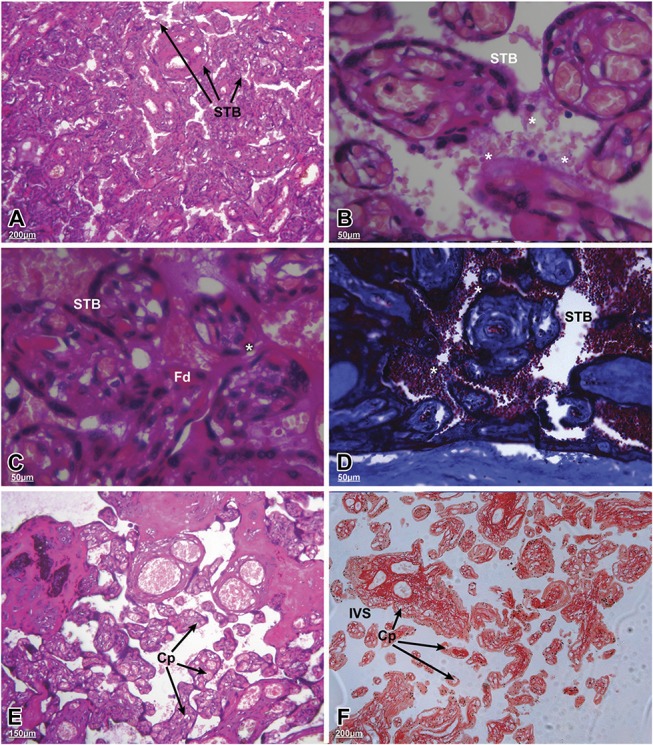
Microstructural changes in the terminal villi of preterm placentas from HIV-positive women on antiretroviral therapy (ART). The sections were stained using hematoxylin and eosin (A–C, E and F) or Masson's trichrome (D). A, General architecture of the floating villi with their syncytiotrophoblast (STB) coverings (arrows; scale bar, 200 µm). B and C, Higher magnification of different fields from the same placenta showing regions of STB delamination associated with the terminal villi (asterisks). C, Areas of perivillous, eosinophilic fibrin deposits (Fd) were evident (scale bar 50 µm). D, Red blood cell adherence to the villi (asterisks; scale bar 50 µm). E and F, Higher number of capillaries (Cp) in the terminal villi with significant increases in the intervening intervillous spaces (IVS) [scale bars: (E) 150 µm; (F) 200 µm]. A, 35 weeks; (B) 35 weeks; (C) 35 weeks; (D) 32 weeks; (E) 33 weeks; and (F) 31 weeks of gestation.

Placentas such as the one shown in Figure 2A were examined at higher magnification (B and C), which revealed syncytiotrophoblast delamination and prominent perivillous eosinophilic fibrin, respectively. In some areas, red blood cell adhesion to the villus surfaces was also evident (Fig. 2D). The terminal villi showed an increase in capillary density and a decrease in villus surface area (Figs. 2E and F).

Compared with HIV-seronegative preterm placentas, those from seropositive women who gave birth during the preterm period had significantly smaller villous areas (2650–3150 µm^2^/random field vs. 2900–3450 µm^2^; *P* < 0.05). The preterm placentas from seropositive women also exhibited a reduction in villus perimeter (mean of 186 vs. 244 µm; *P* < 0.005). The villus surface area (HIV-negative, 2450–2700 µm^2^/random field versus HIV-positive, 2600–2850 µm^2^; *P* = 0.22) and perimeter (HIV-negative, 185 µm vs. HIV-positive, 181 µm) from term placentas did not vary according to serostatus.

## DISCUSSION

In this study of the effects of HIV infection and ART on the preterm placenta, we found that the samples from the HIV-positive women had unique gross anatomical and histological findings indicative of placental damage. Grossly, placentas from the HIV-positive women had an unusually high rate of marginal cord insertion, thrombosis, infarction, and were thinner. Because all the women in this group received ART, it was not possible to determine whether the observed placental pathologies were associated with HIV infection, treatment, or both. Of relevance, some HIV-associated lesions such as chorioamnionitis, deciduitis, placental membrane inflammation, and funisitis have previously been described.^28^ With the World Health Organization (WHO) 2013 “Option B+” policy, all HIV-positive women of reproductive age are either on ART preconception or are initiated immediately if the diagnosis is made during pregnancy.^29^ With ART adherence, viral suppression may be achieved, and it is therefore plausible to suggest that most of the features observed in the placentas of HIV-positive women are associated more with ART than the HIV infection.

As to specific features, the high frequency of marginal cord insertions in HIV-positive women is unusual. Previously, this anatomical feature has been associated with poor fetal outcomes including PTB and intrauterine growth restriction because of chorionic regression and impairment of placental function.^30,31^ The exact mechanism of marginal cord insertion is unknown. This type of insertion may result from a process known as trophotropism in which the chorion frondosum of the early placenta migrates to a more well-vascularized zone of the uterine wall as pregnancy advances.^32^ This feature was observed in both HIV-positive term and preterm placentas with almost equal frequency and may be related to either the HIV-positive serostatus or ART. Further work is needed to decipher the potential mechanisms involved in this process. There were nearly equal numbers of HIV-positive and -negative women with PTB who had fetal membranes with features of chorioamnionitis. However, we did not assess other specific forms of infection in this study. The presence of placental infection increases by 2-fold the risk of PTB.^33–35^ The higher incidence of meconium staining of preterm placental membranes in HIV-positive mothers may also signify fetal distress because of poor placental function including uteroplacental insufficiency.

Our finding of increased placental thrombosis and infarction in HIV-positive mothers is in accord with previous reports^36–38^ and at variance with others.^39,40^ These vascular pathologies have been attributed to HIV-associated upregulation of the extrinsic coagulation pathway with fibrinoid deposition.^41,42^ As to mechanism, upregulation of the coagulation cascade leads to increased thrombin generation,^43^ which triggers fibrin generation and platelet aggregation.^44,45^ The consequences could include a predisposition to preterm rupture of membranes, PTB, intrauterine growth restriction, pre-eclampsia, and fetal demise.^46^

We also noted that the placentas of HIV-positive mothers were thinner than those of their uninfected counterparts. This was at odds with a report that found no difference in placental dimensions with HIV serostatus.^21^ By contrast, cytomegalovirus infection is associated with an increase in placental thickness, but the mechanism is not well-understood.^47^ Several other anatomical features did not differ between the placentas of the HIV-positive and uninfected groups. In addition, the mean weights of term and preterm placentas fell within the standard percentiles for their gestational ages.^48^ This was in contrast to previous reports that the placentas of HIV-positive mothers were smaller and weighed less compared with their uninfected counterparts.^49,50^ These findings suggest that ART may preserve placental growth and, ultimately, weight.

As to the histological analyses, villitis and deciduitis were common in placentas from HIV-positive and uninfected mothers. These pathological features are almost always the result of ascending infection, which may lead to necrosis of the terminal villi and decidua.^51^ Their presence may signify undesirable pregnancy outcomes including PTB. By contrast, lesions that were unique to the placentas of mothers with PTB and HIV infection included fibrinoid deposition with villus degeneration, syncytiotrophoblast delamination, increased red cell adhesion, and hypervascularity of the terminal villi. Although a normal placenta may have a small amount of perivillous fibrinoid due to eddying of the blood, significant fibrin deposition occurs secondary to clotting of maternal blood in the intervillous space.^52^ As a result, the entrapped chorionic villi atrophy.^53^ The underlying mechanism of this process remains poorly understood but could be related to the aforementioned HIV-induced immunological activation of the extrinsic clotting pathway.^54,55^ The observed destruction of the syncytiotrophoblast layer in placentas of these patients could be through HIV-induced, cytokine-mediated inflammatory responses and may imply a compromised feto-maternal interface with attendant risks such as PTB.^56,57^

Some of the pathological features that we observed were specific to the terminal villi. Erythrocyte adhesion to this region has been described in placental malaria and in the placentas of growth-restricted fetuses.^58^ This process, which may be initiated by STB destruction or apoptosis,^59^ leads to local immune complex formation, which may further damage STBs, initiating a vicious cycle that ultimately damages the placenta.^60^

Hypervascularity of terminal villi, defined as excessive numbers of capillaries,^27^ is associated with chronic hypoxia and encountered in other obstetric complications.^61–63^ Recent studies in mice exposed to combined ART throughout pregnancy demonstrated enhanced branching of the placental vasculature with increased numbers of small diameter vessels.^64^ This may indicate that maternal ART promotes this process or new capillary formation. Finally, the reduced villus surface area and perimeter in placentas of HIV-positive mothers with PTB was in keeping with other reports.^65,66^ This manifestation has been attributed to new branching of the intermediate villi, giving rise to multiple terminal villi, a potential compensatory mechanism for a dysfunctional placenta.^67,68^

In conclusion, these results imply that maternal HIV and ART are associated with salient morphological changes in preterm placentas that could contribute to delivery before 37 weeks of gestation. Given the observed hypervascularity, the attendant pathologies may be attributable, in part, to hypoxia. Further research to explore potential mechanisms will help elucidate the pathways that are involved perhaps pointing to interventions for decreasing the risk of PTB among HIV-positive women.
